# Surgical and endovascular cerebral revascularization for cerebral vasculitis with inflammatory vessel stenosis: a case series

**DOI:** 10.1007/s00701-024-06007-z

**Published:** 2024-02-23

**Authors:** Simon Schieferdecker, Julian Caspers, Weiss Daniel, Jan Frederick Cornelius, Sajjad Muhammad

**Affiliations:** 1 Department of Neurosurgery, Medical Faculty and University Hospital Düsseldorf, Heinrich-Heine-University Düsseldorf, Düsseldorf, Germany; 2 Department of Diagnostic and Interventional Radiology, Medical Faculty and University Hospital Düsseldorf, Heinrich-Heine-University Düsseldorf, Düsseldorf, Germany; 3 Department of Neurosurgery, King Edward Medical University, Lahore, Pakistan; 4 Department of Neurosurgery, University of Helsinki and Helsinki University Hospital, Helsinki, Finland

**Keywords:** EC-IC bypass, Stenting, Vasculitis, Stenosis, Giant cell arteritis, Takayasu’s arteritis

## Abstract

Autoimmune vasculitides affect the cerebral vasculature significantly in a considerable number of cases. When immunosuppressive treatments fail to prevent stenosis in cerebral vessels, treatment options for affected patients become limited. In this case series, we present four cases of pharmacoresistant vasculitis with recurrent transient ischemic attacks (TIAs) or stroke successfully treated with either extracranial-intracranial (EC-IC) bypass surgery or endovascular stenting. Both rescue treatments were effective and safe in the selected cases. Our experience suggests that cases of pharmacoresistant cerebral vasculitis with recurrent stroke may benefit from rescue revascularization in combination with maximum medical management.

**Supplementary Information:**

The online version contains supplementary material available at 10.1007/s00701-024-06007-z.

## Introduction

Cerebral vasculitides are a heterogeneous group of diseases characterized by inflammation in the walls of arteries or veins. The Chapel Hill Consensus Conference of Nomenclature of Vasculitides classifies the subtypes of vasculitides according to the size of the affected vessels, where all types, from large to small, can be affected [5]. Cerebral manifestations have been reported with varying incidence in all subtypes of vasculitides. Apart from systemic symptoms of inflammation, cerebral vasculitides can lead to subsequent stenosis or embolic events in the inflamed vessels, affecting a significant number of patients. For instance, the incidence of strokes and TIAs due to vasculitis in Takayasu’s arteritis (TA) is estimated to be around 15%, while in cases of giant cell arteritis (GCA), the incidence ranges between 2 and 7% of cases [3, 8]. The involvement of the cerebral vasculature in systemic vasculitis is often marked by a poor prognosis [2, 9]. The systemic treatment of cerebral vasculitis relies mainly on immunosuppressive agents such as corticosteroids, cytostatic agents, and monoclonal antibodies. However, for the treatment of hemodynamic complications of severe vasculitis no standardized guidelines have been established. Only a few isolated case reports of stenting in cerebrovascular stenosis due to GCA have been published [1, 4, 7, 10], and to our knowledge, there is no literature on extracranial-intracranial (EC-IC) bypass surgery in cerebral vasculitides. Therefore, we describe the first cases of EC-IC bypass in GCA and TA and present our case series on cerebral bypass surgery and endovascular stenting in patients with severe arterial stenosis following cerebral vasculitis.

## Case reports

Between 2010 and 2020, we treated four patients. Two patients underwent EC-IC bypass surgery, while the other two were treated with endovascular stenting. Demographic data for the patients as well as the immunosuppressive medication is given in Table 1. All patients showed disease progression despite maximum medical management, before being suggested for revascularization by the treating physicians.

**Table 1:** Demographic data for the patients as well as the immunosuppressive medication

Patient	Sex	Age at moment of therapy	Disease	Affected vessels	Medication	Surgery/intervention	Follow-up
1	f	38	TA	**ICA occlusion left**, ICA stenosis right, PCA stenosis bilateral	CS, CP	EC-IC bypass bilateral	144 m
2	f	66	GCA	**ICA stenosis bilateral**	CS, MTX	EC-IC bypass bilateral	31 m
3	m	68	GCA	**V4 stenosis left**, V3 occlusion right	CS Tocilizumab	V4 stent left	33 m
4	f	42	SLE	**ICA/M1 stenosis right,** A1 stenosis right	CS	ICA/M1 stent right	29 m

*TA* Takayasu’s arteritis, *GCA* giant cell arteritis, *SLE* systemic lupus erythematosus, *CS* corticosteroids, *CP* cyclophosphamide, *MTX* methotrexate

### Case 1

A 38-year-old female diagnosed with TA experienced global aphasia and right-sided hemiparesis (3/5) after 1 year of immunosuppressive therapy with corticosteroids.

Radiographic imaging revealed bilateral stenosis of both the internal carotid artery (ICA) and posterior cerebral artery (PCA). CT perfusion imaging showed reduced cerebral perfusion in both middle cerebral artery (MCA) territories and multifocal strokes in the PCA territories. Acetazolamide challenge showed exhausted cerebrovascular reserves bilaterally. Subsequent angiography revealed inflammatory changes but no relevant stenosis in the superficial temporal arteries (STA). After interdisciplinary discussion, we recommended EC-IC bypass surgery as rescue treatment due to the length of the stenosis and multiple small passages of total occlusion. We performed bilateral standard STA-M4 EC-IC bypass surgery, and the immunosuppressive therapy was escalated. Postoperatively, the patient’s symptoms markedly improved, with only mild remaining hemiparesis. During the 10-year follow-up no inflammation and subsequent stenosis occurred in the bypass vessels. No further TIAs or strokes occurred during follow-up.

### Case 2

A 66-year-old female diagnosed with GCA suffered transient ischemic attacks (TIAs) in the right MCA territory after 1 year of corticosteroid therapy. Computed tomography perfusion imaging (CT-P) and angiography (CT-A) revealed bilateral ICA stenosis with corresponding reduced perfusion in the MCA territories, with significantly worse perfusion on the right side. Acetazolamide challenge showed an exhausted cerebrovascular reserve on the left side and a paradoxical reaction on the right side. After interdisciplinary discussion, EC-IC bypass was chosen as the therapy. We performed a standard STA-M4 bypass on the right side. On the left side, however, the STA was severely stenotic due to the vasculitis. Therefore, we used the patent occipital artery (OA) and performed an OA-M4 bypass on the left side. Postoperative the patient had no further cerebrovascular incidents. Notably, the right STA that was used for the bypass did not show signs of inflammation during the follow-up.

### Case 3

**Fig. 1:**
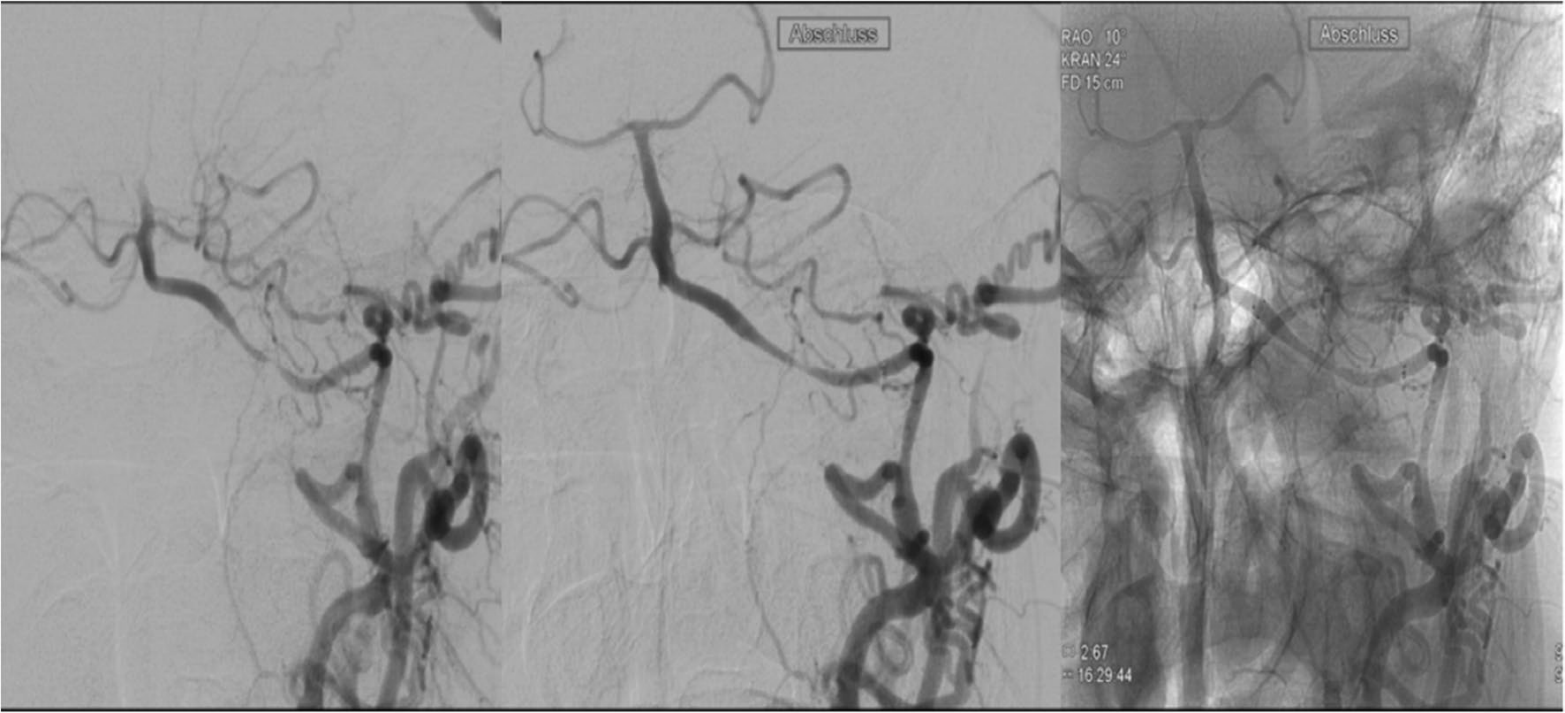
Case 3—Digital substraction angiography (DSA) of the posterior circulation with magnification in RAO 10° and CRAN 24° pre-stenting (**a**) and post-stenting (**b**). Unsubstracted DSA control image with the same image parameters post-stenting (**c**). An opacification of prominent cervical branch collaterals by contrast reflux is seen

**Fig. 2:**
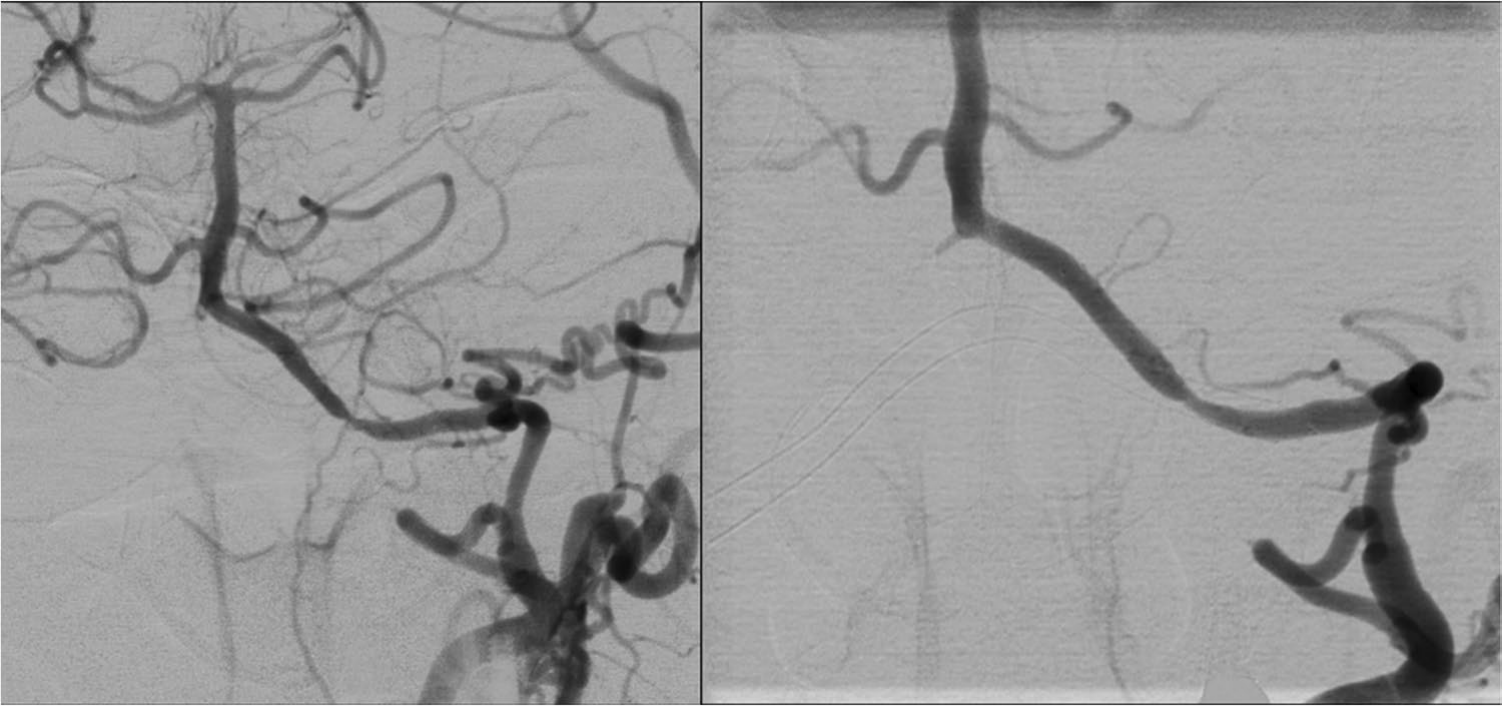
Case 3—Digital substraction angiography (DSA) of the posterior circulation in RAO 6° and CRAN 12° with magnification after 5 months (**a**) and after 12 months (**b**). Both images revealed a re-stenosis of the left vertebral artery

A 68-year-old male presented with visual disturbances. Diagnostic imaging revealed multiple small strokes in the territory of the posterior circulation. GCA was diagnosed as the cause of the corresponding left-sided vertebral artery (VA) stenosis in the V4 segment and the right-sided VA occlusion in the V3 segment according to standard diagnostic criteria. The patient received corticosteroids and monoclonal antibodies as treatment. After interdisciplinary discussion, stenting of the left VA stenosis was chosen. Stenting of the V4 segment was performed using a self-expanding laser-cut stent without further balloon angioplasty, leaving a 50% stenosis. Routine digital subtraction angiography (DSA) follow-up 5 months after stenting revealed a slightly progressing restenosis within the left VA stent. Another DSA 1 year after stenting showed regression of the restenosis to 40% of the lumen. Immunosuppressive therapy was escalated, and the stenosis proved stable in all subsequent follow-ups. No further TIAs or strokes occurred during the follow-up. Figure 1 shows the initial stenosis as well as post-stenting image. Figure 2 shows the subsequent restenosis at 5 months and 1-year follow-up.

### Case 4

**Fig. 3:**
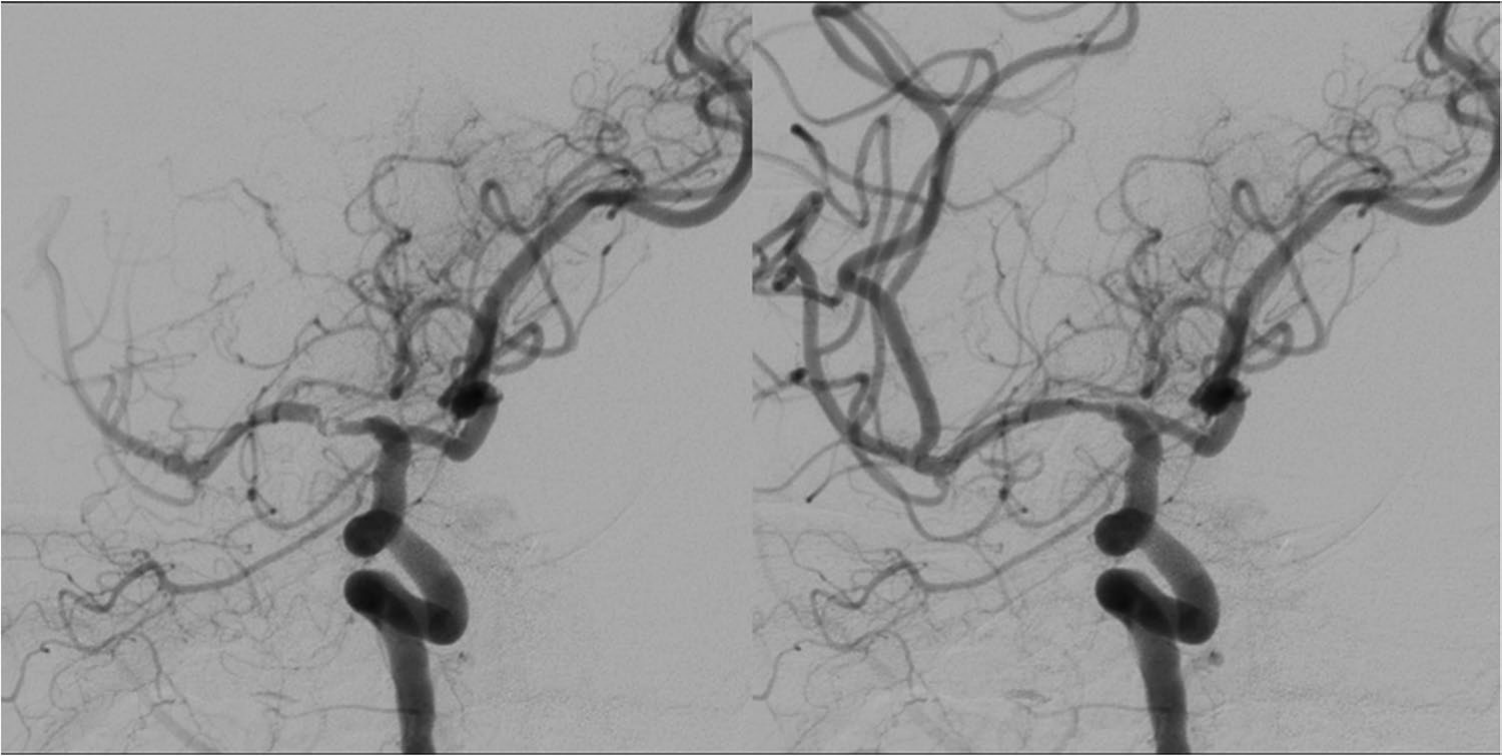
Case 4—Digital substraction angiography (DSA) of the middle cerebral artery territory in LAO 36° and CRAN 24° with magnification pre-stenting (**a**) and post-stenting (**b**)

A 42-year-old female patient with systemic lupus erythematosus (SLE) presented with right-sided MCA and anterior cerebral artery (ACA) hypoperfusion (diagnosed on CT perfusion imaging) leading to severe left-sided hemiparesis (1/5) and aphasia. Cranial computed tomography revealed partial infarction within the right MCA territory with large areas of hypoperfusion in the right MCA and ACA territories beyond the infarction core, i.e. penumbra tissue. DSA and MR imaging revealed vasculitis in the cavernous segment of the ICA with subsequent stenosis. After interdisciplinary discussion, endovascular stenting of the distal ICA and M1 segment was performed with a self-expanding laser-cut stent without the use of balloon angioplasty. The patient’s aphasia fully recovered and hemiparesis markedly improved to a grade of 4/5. During the follow-up no further TIAs or strokes occurred. Figure 3 shows images of the stenosis before and after stenting.

## Discussion

The treatment of steno-occlusive disease of the cerebral vasculature through endovascular interventions or bypass surgery is well established. However, the literature on cases with non-atherosclerotic genesis is scarce. Cerebral autoimmune vasculitis is a rare disease with potential devastating complications, particularly in pharmacoresistant cases. Here, we present four cases of vasculitis treated by cerebral revascularization with EC-IC bypass surgery or endovascular stenting as rescue treatments. Despite maximum medical management, all presented cases displayed symptoms of progressive stroke. It is important to note that the presented rescue treatments are not approved standardized therapies for vasculitis. As such, several aspects warrant discussion. The main questions we encountered when discussing the abovementioned patients were: (1) are surgery or endovascular interventions in the inflamed vessel segments safe? (2) will the procedure trigger inflammation, potentially spreading to the bypass vessel or stented segment? (3) might the surgery or intervention have only temporary efficacy, with the natural course of the disease ultimately obliterating the bypass vessels or stent in the long term? Regarding question (1), our short case series involved the implantation of two stents in patients with signs of active disease and inflammation. Both stents were placed in the inflamed segments, and no complications such as vessel rupture, in-stent thrombosis, or thromboembolic events occurred. Bypass surgery was performed in one patient with giant cell arteritis where the STA was inflamed but not stenotic. Intraoperatively, the inflammation had no adverse effect on the surgical procedure, including suturing, or the durability of the vessel, and there were no postoperative complications regarding the patency of the STA. Addressing question (2), our series included bilateral EC-IC bypasses in a patient with TA and inflamed but non-stenotic STAs. Postoperatively, no reactive exacerbation of the inflammation with subsequent stenosis of the operated vessels was observed. Furthermore, when utilizing the uninflamed OA as a donor vessel in a patient with GCA, there was no triggering of vasculitis in the utilized vessel. Concerning question (3), we implanted a stent into the inflamed VA lumen in case 3. Within 5 months, the patient exhibited a slightly progressive, asymptomatic restenosis within the stent. Following the escalation of the immunosuppressive medication, the stenosis stabilized, and no further activity was observed in the affected segment. For all other cases, we did not observe any long-term restenosis or additional stenosis indicative of progressive disease in the treated vessels. Our findings are consistent with the already reported cases of rescue endovascular stenting in GCA documented in the literature [1, 4, 7, 10]. When bypass surgery is considered and the stenotic vessels are the large intracranial vessels of the skull base, then standard STA-M4 bypasses will most often not include the inflamed vessels. Even so, in the presented case of GCA that we operated on, we could demonstrate the safety in surgically employing the inflamed STA for bypass surgery. Nakayama et al. have treated a case of cerebral vasculitis due to graft-versus-host disease in allogenic bone marrow transplant. In the case report, a patient with TIAs due to vasculitis in the territory of the MCA received an STA-MCA bypass. Notably, the authors did neither record any triggered inflammation in the surgically manipulated vessels nor did inflammation spread to the bypass within the documented follow-up [6].

This study has several limitations. Even including the already published cases of stenting in GCA, there is still only a handful of cases available. Consequently, the conclusions drawn in terms of safety and efficacy, particularly regarding the durability of inflamed vessels for intervention and surgery, need to be further validated with larger cohorts. Additionally, if the grade of inflammation’s relevance is hypothesized not to be a significant factor in the choice of treatment, the decision of bypass surgery or stenting should be made based on stringent selection criteria, involving detailed perfusion studies and the quantification of cerebrovascular reserve capacity, as in other stenoocclusive conditions like moya moya disease. Furthermore, long-term success of such rescue treatments needs to be verified as we can only present one case with a 10-year follow-up. For all other patients, the follow-up duration ranges from 29 to 33 months.

## Conclusion

In conclusion, both EC-IC bypass surgery and endovascular stenting appear to be safe and effective treatment options for cases of acute cerebral vasculitis with subsequent stenosis. Our limited experience demonstrates that cases of pharmacoresistant cerebral vasculitis with recurrent stroke may benefit from rescue revascularization combined with maximal medical management. Thus, the critical patient selection and interdisciplinary management of such cases remain imperative. The interventions, even in inflamed vessels, were found to be safe and devoid of complications.

## Supplementary Information

Below is the link to the electronic supplementary material.

Supplementary file1 (DOCX 13 KB)

## Data Availability

All relevant patient data is given in the manuscript. Information regarding the devices used during interventions or any local therapy regiments (i.e. platelet inhibition regimen and duration) can be accessed via the supplementary material.
