# Supplementary material for: Surgical and endovascular cerebral revascularization for cerebral vasculitis with inflammatory vessel stenosis: a case series
Source: Acta Neurochir (Wien). 2024 Feb 23;166(1):103. doi: 10.1007/s00701-024-06007-z (PMC10891259; doi:10.1007/s00701-024-06007-z)
Supplement: Supplementary file 1 — Supplementary file1 (DOCX 13 KB) [file 701_2024_6007_MOESM1_ESM.docx]

**SUPPLEMENTARY MATERIAL**

**Catheter systems and device sizing**

**Case 3:**

Catheter: 6F guiding catheter (ENVOY™ MPD, Codman Neurovascular, Inc., Boston, MA, USA), 5F intermediate catheter (SOFIA™, MicroVention Inc., Aliso Viejo, CA, USA), 0.0165-inch microcatheter (NeuroSlider® 17, Acandis GmbH, Pforzheim, Germany)

Stent: 4 mm x 30 mm ACCLINO® flex plus Stent (Acandis GmbH, Pforzheim, Germany).

**Case 4:**

Catheter: 7F guide catheter, 5F intermediate catheter (NAVIEN™ A+, Medtronic plc, Dublin, Ireland) 0.021-inch microcatheter (NeuroSlider® 21, Acandis GmbH, Pforzheim, Germany)

Stent: 5.5 mm x 25 mm ACCLINO® flex plus Stent (Acandis GmbH, Pforzheim, Germany).

**Platelet inhibition regimen**

In our clinic all patients initially receive weight-adapted tirofiban for platelet inhibition, followed by a switch to clopidogrel. Effectivity is tested by means of flow cytometric analysis of vasodilator-stimulated phosphoprotein (VASP test). Clopidogrel is then administered for 3 months. In addition to that, all patients receive a lifelong therapy with 100mg aspirin daily.
